# Does the kappa opioid receptor system contribute to pain aversion?

**DOI:** 10.3389/fphar.2014.00253

**Published:** 2014-11-17

**Authors:** Catherine M. Cahill, Anna M. W. Taylor, Christopher Cook, Edmund Ong, Jose A. Morón, Christopher J. Evans

**Affiliations:** ^1^Department of Anesthesiology and Perioperative Care, University of California IrvineIrvine, CA, USA; ^2^Department of Pharmacology, University of California IrvineIrvine, CA, USA; ^3^Department of Biomedical and Molecular Sciences, Queen’s UniversityKingston, ON, Canada; ^4^Semel Institute for Neuroscience and Human Behavior, University of California Los AngelesLos Angeles, CA, USA; ^5^Department of Anesthesiology, Columbia University Medical Center, New York, NYUSA

**Keywords:** kappa opioid receptor, pain, aversion, reward system ventral tegmental area, dopamine, negative reinforcement

## Abstract

The kappa opioid receptor (KOR) and the endogenous peptide-ligand dynorphin have received significant attention due the involvement in mediating a variety of behavioral and neurophysiological responses, including opposing the rewarding properties of drugs of abuse including opioids. Accumulating evidence indicates this system is involved in regulating states of motivation and emotion. Acute activation of the KOR produces an increase in motivational behavior to escape a threat, however, KOR activation associated with chronic stress leads to the expression of symptoms indicative of mood disorders. It is well accepted that KOR can produce analgesia and is engaged in chronic pain states including neuropathic pain. Spinal studies have revealed KOR-induced analgesia in reversing pain hypersensitivities associated with peripheral nerve injury. While systemic administration of KOR agonists attenuates nociceptive sensory transmission, this effect appears to be a stress-induced effect as anxiolytic agents, including delta opioid receptor agonists, mitigate KOR agonist-induced analgesia. Additionally, while the role of KOR and dynorphin in driving the dysphoric and aversive components of stress and drug withdrawal has been well characterized, how this system mediates the negative emotional states associated with chronic pain is relatively unexplored. This review provides evidence that dynorphin and the KOR system contribute to the negative affective component of pain and that this receptor system likely contributes to the high comorbidity of mood disorders associated with chronic neuropathic pain.

## INTRODUCTION

Chronic pain may be considered an epidemic in many westernized countries affecting 25% of the population, and where quality of life of chronic pain patients is reported to be lower than other disorders such as heart failure, renal failure and even depression (O’Connor, 2009). Pain is a multidimensional experience comprised of sensory, cognitive, and emotional (subjective) components, which are processed within discreet but interacting brain structures. Many chronic pain states are accompanied by dramatic sensory disturbances that result in pain hypersensitivity (allodynia and hyperalgesia) and tonic (unprovoked) ongoing pain. However, the negative affect, or how much the pain is ‘bothersome’ significantly impacts the quality of life of the sufferer. Notably, the emotional component of pain has been argued to be a greater metric of quality of life than its sensory component, and thus understanding the processes that influence this pain characteristic is essential to developing novel treatment strategies.

Neuroplasticity in brain regions important for the expression of affect may underlie the comorbidity between chronic pain and Axis I disorders of the DSM-V, including depression, anxiety disorders, bipolar disorder, and ADHD. Comorbidities with each of these disorders in chronic pain patients have has been well documented, where depression is the most common comorbidity, with some studies finding a prevalence rate approaching 100% among clinical chronic pain samples (reviewed by Nicholson and Verma, 2004). In fact, chronic pain is second only to bipolar disorder as the major cause of suicide among all medical illnesses, further highlighting the importance of negative affect in this condition (Juurlink et al., 2004; Asmundson and Katz, 2009; Elman et al., 2013). Nevertheless, it remains debated whether mood disorders are a consequence of, or a pre-existing susceptibility for the genesis of chronic pain (Von Korff et al., 1993; Fishbain et al., 1997; Blackburn-Munro and Blackburn-Munro, 2001; Miller and Cano, 2009). Clinical studies specifically aimed at identifying risk factors that may predict the incidence of or transition to chronic pain are now being pursued (Attal et al., 2014; Mundal et al., 2014).

Dysfunction of reward mesolimbic circuitry underlies the etiology of many psychiatric disorders, including depression. Because it is common for chronic pain to be comorbid with diseases known to have deficits in the dopamine mesolimbic system, it is posited that this dysfunction also contributes to the genesis of chronic pain (Taylor, 2013; Cahill et al., 2014). For example, a high prevalence of chronic pain is common in disorders linked with deficits in the dopamine system, including disorders of mood and affect, substance abuse, and Parkinson’s disease (Jarcho et al., 2012). The statistic that substance abusers are six times more likely to develop chronic pain than its prevalence in society (Gureje et al., 1998; Verhaak et al., 1998; Jamison et al., 2000; Rosenblum et al., 2003) is not surprising, if dysfunction of mesolimbic reward system contributes to chronic pain states. In contrast, clinical conditions associated with elevated mesolimbic dopamine (e.g., schizophrenia) have higher pain thresholds (Dworkin, 1994; Boettger et al., 2013). It should be noted that an alternative explanation for the increased prevalence of chronic pain in substance abusers is the occurrence of opioid-induced hyperalgesia. Opioid-induced hyperalgesia is a paradoxical increase in pain sensitivity following opioid administration via either chronic exposure [e.g., morphine, hydrocodone, oxycodone and methadone, or single exposure (e.g., Remifentanil) Chu et al., 2008; Lee et al., 2011; Fletcher and Martinez, 2014]. Studies have identified various mechanisms that may account for the occurrence of opioid-induced hyperalgesia including sensitization of pro-nociceptive pathways caused by long term potentiation of synapses between nociceptive C fibers and spinal dorsal horn neurons (Drdla et al., 2009) and neuroimmune responses reducing GABAergic inhibition (Ferrini et al., 2013).

Pain and reward are considered opponent processes but are processed within overlapping or interacting brain structures (e.g., anterior cingulate cortex, dorsal and ventral striatum, and amygdala). It has been demonstrated that rewarding stimuli such as food and pleasurable music decrease pain sensitivity (Leknes and Tracey, 2008), whereas pain can impair reward processing, which can lead to an anhedonic state (Marbach and Lund, 1981; Nicholson and Verma, 2004; Elman et al., 2013). Canonical neurotransmitters involved in affect and reward are dopamine, serotonin, norepinephrine, and endogenous opioids. Modulating the function of these neurotransmitters is associated with altered mood states. The mesolimbic system, which includes the ventral tegmental area (VTA) and the nucleus accumbens (NAc, part of the ventral striatum), is responsible for the expression of positively motivated behaviors and reinforcement learning produced by natural and drug rewarding stimuli (Fields et al., 2007; Sun, 2011). Few studies have examined dysfunction of this circuitry in chronic pain, and whether the mesolimbic dopaminergic system contributes to the aversive component of ongoing persistent pain. Some clues have emerged from functional imaging studies on healthy volunteers and chronic pain patients. Functional magnetic resonance imaging studies of clinical pain cohorts demonstrate altered connectivity between the mesolimbic system and various cortical structures (Apkarian et al., 2005; Jensen et al., 2013; Ichesco et al., 2014). For example, greater functional connectivity of the NAc with the prefrontal cortex predicted pain persistence, implying that corticostriatal circuitry is causally involved in the transition from acute to chronic pain (Baliki et al., 2012). Functional connectivity analysis in neuropathic pain animals also revealed that changes in connectivity were primarily (97%) localized within the limbic system (NAc, septum and ventral pallidum, amygdala and hippocampus), as well as between the limbic and nociceptive systems (thalamus, primary sensory cortices, insula cortex, and periaqueductal gray; Baliki et al., 2012, yet no connectivity changes were observed within the nociceptive network). A corollary study in patients reported that chronic back pain patients exhibited brain activity in regions responsible for emotion-related circuitry, whereas acute back pain patients demonstrated activity in nociceptive circuitry (Hashmi et al., 2013). These studies suggest that the limbic system is engaged in clinical and experimental models of chronic pain. It is unknown how or why greater functional connectivity with limbic structures contributes to chronic pain, although this system is likely engaged to modulate the affective component of pain and gives salience to the pain experience via release of dopamine. The fact that dopamine release in the ventral striatum is associated with placebo-induced analgesia and anticipation of analgesia (Scott et al., 2008; Tracey, 2010; Abhishek and Doherty, 2013) also suggests that dopamine release in the mesolimbic system may be important in modulating the negative affect component of pain. The interplay between reward pathways and pain validate the importance of this circuitry, not only in the chronicity of pain, but also the lack of opioid effectiveness in treating chronic pain (including that of neuropathic origin).

Opioids and their receptors play a central role in various physiological effects throughout the peripheral and central nervous systems. In addition to their ability to modulate the sensory component of pain (the intensity), opioids also modulate the emotional, aversive component of pain (affective, unpleasant component). For example, a patient being treated with opioids for post-operative pain may still feel the sensory component of pain, but it no longer bothers them. There is strong evidence that release of dopamine within the ventral striatum is responsible for the mood altering properties of opioids. However, opioid-evoked release of dopamine also contributes to their abuse potential, where an allostatic shift in reward signaling leads to the pathological state of addiction. Mu opioid receptor (MOR) agonists positively modulate mood and are the predominant opioid drugs used for clinical and recreational purposes. However, both delta (DOR) and kappa opioid receptors (KORs) also modulate mood and emotion, but in opposite directions (Lutz and Kieffer, 2013). Activation of the KOR causes dysphoria (defined as unpleasant or profound feeling of unwell/unease) in humans and an aversive response in animals, evidenced by its ability to produce a conditioned place aversion in animals (Land et al., 2009; Tejeda et al., 2013). One of the underlying mechanisms thought to account for the dysphoric effects of KOR drugs is their ability to suppress mesolimbic dopamine release within reward circuitry. This review will posit that disruption in mesolimbic cortical circuitry plays an important role in chronic pain and that activity at the KOR is an important regulator of this circuitry. It will also highlight inferences that this opioid receptor contributes to the high incidence of mood disorder comorbidity in various chronic pain states.

## THE VENTRAL TEGMENTAL AREA IS A CENTRAL LOCUS FOR PAIN AND PLEASURE

A decrease or suppression of mesolimbic dopaminergic transmission that originates in the VTA is one mechanistic commonality between a stress response, the precipitation of an aversive state, and chronic pain. Salience is one of the key functions of the mesolimbic dopaminergic circuitry that is encoded via interactions between tonic and phasic spikes in dopamine neurons (McClure et al., 2003). The ‘pain neuromatrix’ has been described as a salience network where the neurocircuitry related to emotion rather than the sensory aspects of pain are considered to have salient value (Legrain et al., 2011; Mouraux et al., 2011). It was recently hypothesized that aberrant functioning of the brain circuits which assign salience values to stimuli may contribute to chronic pain (Borsook et al., 2013). We will focus the discussion on the circuitry of inputs and outputs of midbrain dopaminergic neurons, as this neurocircuitry is engaged by salience attributed to a range of stimuli, including pain (Berridge, 2007; Leknes and Tracey, 2008; Elman et al., 2013). Moreover, this system is engaged by punishment and contributes to negative reinforcement learning (i.e., removal of a negative stimulus, including pain, is rewarding). Alterations in dopamine signaling are associated with motivational deficits, and animals in chronic pain show impaired motivated responses to natural and drug reward (Navratilova and Porreca, 2014; Schwartz et al., 2014). The motivational effect for place preference of analgesic drugs hypothesized to reflect the rewarding component of pain relief is currently being used to assess the affective or tonic-aversive component of pain. Magnussen et al. (2009) were the first to report that non-rewarding analgesics produce a place preference in chronic pain, but not in pain-naïve animals. Subsequently, King et al. (2009) reported that intrathecal lidocaine produced a place preference in an animal model of neuropathic pain, but not in pain-naïve animals. Many studies have now used this paradigm to understand the mechanisms underlying the tonic-aversive component of pain (De Felice et al., 2013; Cahill et al., 2014; Roughan et al., 2014; Xie et al., 2014), which is predicted to have construct validity for screening novel analgesic drugs for clinical development. Analgesic place preference was blocked by intra-NAc injections of dopamine receptor antagonists (Navratilova et al., 2012), suggesting that dopamine release is important for the expression of negative reinforcement associated with pain relief. A clinical correlate to these studies has been described whereby reciprocal negative/positive signals in the NAc correlated with pain onset/offset, respectively (Becerra and Borsook, 2008). Additionally, negative correlations between pain and mesolimbic dopamine activity in humans has been described (Borsook et al., 2007; Wood et al., 2007; Jarcho et al., 2012). It is worth noting that there is no evidence that non-rewarding drugs that produce negative reinforcement in models of chronic pain become rewarding after prolonged use, (i.e., produce psychological dependence). Evidence against this argument is the lack of reported dependence for non-rewarding analgesics including local anesthetic patches, clonidine or tricyclic antidepressants used to manage pain in various clinical pain populations.

**FIGURE 1:**
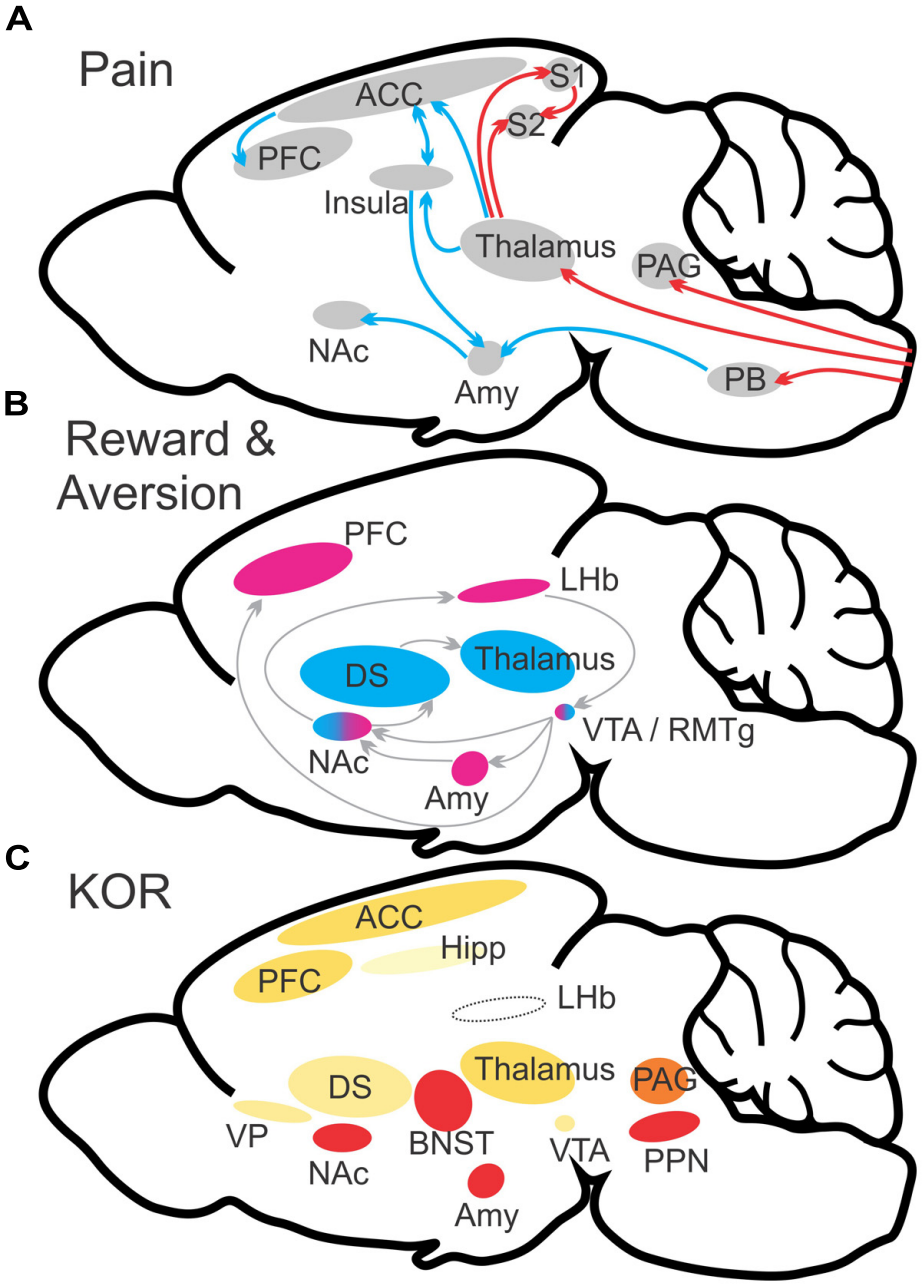
**Schematic illustration of major brain networks involved in pain and reward processes (A,B). (A)** Ascending projections convey pain signaling to multiple brain structures including the periaqueductal gray, thalamus, and parabrachial nucleus. Continued and distinct processing occurs for both sensory (red arrows) and affective (blue arrows) dimensions of pain perception. **(B)** Reward/aversion processes involve multiple overlapping and interacting networks. Shown are the major structures involved in reward (blue) and aversion (magenta). **(C)** KOR abundance in relevant brain structures. Heat map color-coded (Red = most abundant) by reported radioligand binding (Mansour et al., 1987; Le Merrer et al., 2009). ACC, anterior cingulate cortex; Amy, amygdala; BST, bed nucleus of the stria terminalis; DS, dorsal striatum; LHb, lateral habenula; NAc, nucleus accumbens; OFC, orbitofrontal cortex; PAG, periaqueductal gray; PB, parabrachial nucleus; PFC, prefrontal cortex; PPN, pedunculopontine nucleus/pedunculopontine tegmental nucleus; S1, primary somatosensory cortex; S2, secondary somatosensory cortex; VP, ventral pallidum; VTA, ventral tegmental area. **(A,B)** adapted from Cahill et al. (2014).

The VTA is the origin of dopaminergic neurons within the mesocorticolimbic system that mediates reward, motivation, and arousal. There are various inputs to the VTA that result in the inhibition of VTA dopaminergic neurons and are attributed to the expression of an aversive state (**Figure 1**). These brain structures include the habenula, rostromedial tegmental nucleus (RMTg), and ventral pallidum. The habenula is a small brain structure located near the pineal gland and the third ventricle, sometimes called the tail of the VTA. Recent reviews highlight the critical role this brain structure has in influencing the brain’s response to pain, stress, anxiety, sleep, and reward (Shelton et al., 2012; Velasquez et al., 2014). The habenula evaluates external stimuli and directs the motivation of appropriate behavioral response, thereby contributing to reward-related learning to reinforce or avoid actions based on previous outcomes. It primarily contains GABAergic neurons that control activity of the VTA, substantia nigra, locus coeruleus, and raphe nucleus. The RMTg is a midbrain structure located at the caudal tail of the VTA. Its function is to convey salient positive and negative signals to dopamine neurons and participate in appetitive behavioral responses (Bourdy and Barrot, 2012). The ventral pallidum is a brain structure within the basal ganglia located along the external segment of the globus pallidus. It projects to the VTA (Haber et al., 1985), subthalamic nucleus, thalamus, and lateral hypothalamus, and has reciprocal projections to the ventral striatum (including the NAc). It is part of the striatopallidal indirect cortico-basal ganglia pathway that regulates emotion, motivation, and movement. The periaqueductal gray (PAG) also projects directly to the VTA, providing the third heaviest subcortical source of glutamate input to the VTA (Geisler et al., 2007) synapsing onto both gamma-aminobutyric acid (GABA) and dopaminergic neurons (Omelchenko and Sesack, 2010). Based on its functions, the PAG is likely to supply VTA neurons with information important for processing nociceptive signals, defensive and stress behaviors, and rewarding responses to opiates.

**FIGURE 2:**
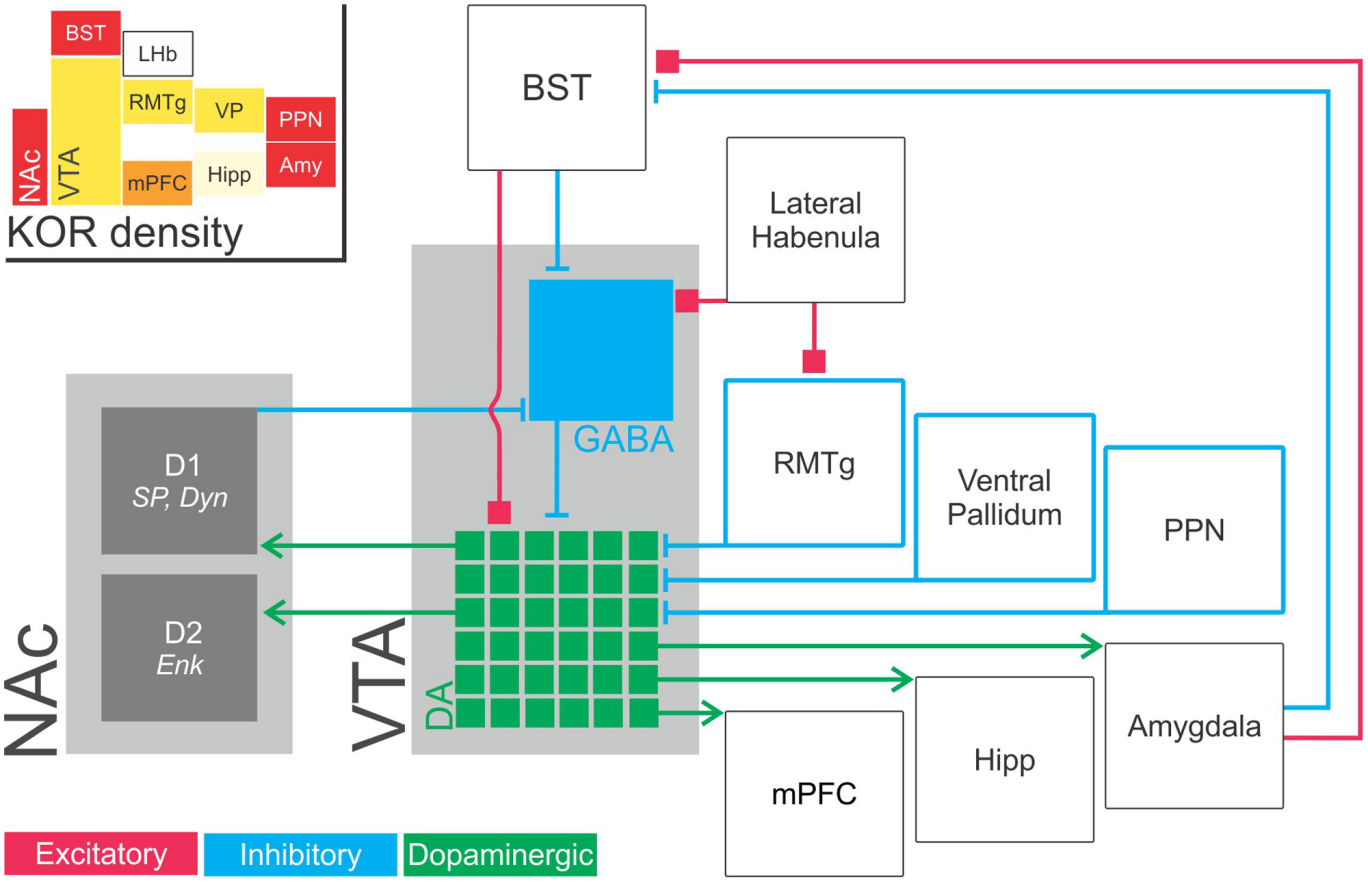
**Schematic illustration of inputs to and outputs from VTA dopaminergic neurons.** VTA dopaminergic neurons project to the NAc (required for responding to reward prediction cues), medial prefrontal cortex (implicated in working memory and attentional processes), basolateral amygdala (implicated in emotion, reward, fear conditioning, and avoidance) and hippocampus (memory; green, dopaminergic components). Notably, VTA dopaminergic outputs are extensively differentiated with distinct projections from specific populations of dopaminergic neurons to specific downstream structures. In this way, mesolimbic dopaminergic activity functions in seemingly contradictory processes such as reward and aversion (McCullough et al., 1993; Stevenson et al., 2003; Baliki et al., 2010; McCutcheon et al., 2012). That is, the functions of ventral tegmental area dopaminergic projections is determined by network topology rather than simply by the choice of neurotransmitter. VTA dopaminergic neurons receive dense GABAergic modulatory input from numerous extrinsic structures and from GABAergic interneurons within the ventral tegmental area itself (blue). GABAergic projections to the VTA has been identified from the NAc (Nauta et al., 1978); ventral pallidum (Haber et al., 1985); RMTg (Jhou et al., 2009) and the pedunculopontine tegmental nucleus/lateral dorsal tegmentun (Omelchenko and Sesack, 2005; Good and Lupica, 2009). These neurons are also modulated by excitatory inputs (red) from the bed nucleus of the stria terminalis (Georges and Aston-Jones, 2001, 2002; Watabe-Uchida et al., 2012). (Inset) KOR abundance in these structures. Heat map color-coded (Red = most abundant) by reported radioligand binding (Mansour et al., 1987; Le Merrer et al., 2009). Amy, amygdala; BST, bed nucleus of the stria terminalis, D1, dopamine receptor D1; D2, dopamine receptor D2; Enk, enkephalin; Hipp, hippocampus; LHb, lateral habenula; mPFC, medial prefrontal cortex; NAc, nucleus accumbens; PPN, pedunculopontine nucleus/pedunculopontine tegmental nucleus; RMTg, rostromedial tegmental nucleus; VP, ventral pallidum; SP, substance P; VTA, ventral tegmental area.

Gamma-aminobutyric acid is the primary neurotransmitter in RMTg neurons that project to the VTA (Jhou et al., 2009). Activation of these neurons release GABA directly on VTA dopamine neurons leading to suppression of dopaminergic transmission. Functionally, when the RMTg is surgically lesioned, the response to aversive stimuli is attenuated, which suggests a convergence of aversive inputs within the RMTg (Jhou et al., 2009). The habenula is another input to the VTA that suppresses VTA dopaminergic transmission, and it does so via a direct and indirect pathway (Omelchenko and Sesack, 2010). The habenula is divided into medial and lateral (LHb) components that have different afferent and efferent connections (Velasquez et al., 2014). The LHb is topographically organized with the medial division sending excitatory glutamatergic projections to the VTA that synapse on GABAergic interneurons (Ji and Shepard, 2007; Gonçalves et al., 2012). Activation of this pathway leads to an increase in inhibitory postsynaptic currents in dopamine neurons. The lateral division of the LHb sends excitatory projections to the GABAergic neurons in the RMTg (Gonçalves et al., 2012). Hence, LHb glutamatergic terminals in the RMTg excite GABAergic neurons that in turn synapse with VTA dopaminergic neurons, resulting in an inhibition of dopaminergic neuronal firing (**Figure 2**).

One prominent feature of the habenula is that it is involved in the processing of aversive information, including pain. In addition, repeated or continuous stress can lead to expression of depression-like behavior and exacerbate chronic pain. Importantly, sensitization of the LHb-dopamine circuitry occurs in depressive states (Hikosaka, 2010). Indeed, humans with depression or animal models of depression exhibit hyperactivity within the LHb (Caldecott-Hazard et al., 1988; Morris et al., 1999). Whether the LHb exhibits hyperactivity in chronic pain and contributes to the high comorbidity of mood disorders with chronic pain states remains unexplored. Pain transmission directly and indirectly activates the habenula. Reports show that an aversive stimulus increases the LHb excitatory drive onto GABAergic RMTg neurons (Jhou et al., 2009; Hong et al., 2011; Stamatakis and Stuber, 2012), leading to a decrease in dopamine output (Ji and Shepard, 2007; Matsui and Williams, 2011). The spinal cord projects to the Lhb directly (Craig, 2003) or indirectly via the lateral hypothalamus (Dafny et al., 1996), another brain region well established to modulate pain. Studies using anterograde tracing identified that while spinal cord lamina I nociceptive neurons project primarily to thalamic nuclei, some terminals were found in the dorsomedial hypothalamus (Craig, 2003). The deep dorsal spinal cord projects to the thalamus, globus pallidus, substantia innominata, amygdala, and hypothalamus (Gauriau and Bernard, 2004), and many of these structures influence mesolimbic dopamine circuitry.

Because the habenula is such as small brain structure, imaging studies to examine changes in activity within this region are challenging. Nevertheless, the habenula circuitry is proposed to undergo neuroplastic changes in chronic pain (Shelton et al., 2012), where the hedonic deficit due to dysfunction of reward systems generates a facilitation of pain. Several lines of evidence suggest that LHb neurons are hyperactive in individuals with depression. Such studies led to the successful use of deep brain stimulation (DBS) to manipulate the activity of the habenula as a treatment of major depression (Sartorius and Henn, 2007; Hauptman et al., 2008; Sartorius et al., 2010). The positive outcomes are thought to result from the ability of DBS to suppress the abnormally elevated activity of the habenula. Interestingly the habenula has one of the richest MOR expression patterns in the brain (Zastawny et al., 1994; Bunzow et al., 1995; Kitchen et al., 1997). Morphine injection into the habenula produces analgesia (Cohen and Melzack, 1986; Darcq et al., 2012), and intra-habenular injection of the opioid antagonist naloxone blocks the analgesic effects that result from an injection of morphine into the PAG (Ma et al., 1992). Taken together, pain modulatory systems likely engage this structure for the expression of pain affect.

It cannot be assumed that the inputs to the VTA discussed above result in modulation of the dopaminergic projections to the NAc implicated in reward. The VTA-NAc projection is also implicated in the pathogenesis of stress-related behaviors. Dopaminergic neurons within the VTA project to various brain structures, including the medial prefrontal cortex (mPFC), amygdala, and hippocampus, as well as the NAc. Importantly, there is evidence that discrete subpopulations of VTA dopaminergic neurons exclusively project to only one of these regions and that they are engaged by different stimuli and inputs (Volman et al., 2013). It is well accepted that activation of the VTA dopaminergic neurons projecting to the NAc produces reward-like behavior. Thus, it is not unexpected that aversive stimuli strongly inhibit VTA dopamine neurons (Ungless et al., 2004; Hong et al., 2011; Tan et al., 2012), and optogenetic activation of VTA GABAergic neurons or inhibition of VTA dopaminergic neurons produces a conditioned place aversion (Tan et al., 2012). Similarly, dopamine neurons in the caudal VTA increase firing to aversive stimuli such as a foot shock (Brischoux et al., 2009). However, various studies have demonstrated that salient but aversive stimuli, restraint stress, or even social defeat stress will increase VTA dopaminergic transmission (Anstrom and Woodward, 2005; Anstrom et al., 2009). Studies using fast scan cyclic voltammetry and microdialysis have shown elevated dopamine output in the NAc and mPFC in response to aversive stimuli (Bassareo et al., 2002; Budygin et al., 2012). Although, a recent study in non-human primates argues that aversion does not cause dopamine release (Fiorillo, 2013). Ventral tegmental area dopaminergic projections to other areas such as the mPFC and amygdala are also involved in stress-related behaviors. The mPFC both receives dopaminergic projections from the VTA and sends projections back to the VTA and the NAc, thus forming a regulatory feedback system (Nestler and Carlezon, 2006). An elegant study recently reported that activation of laterodorsal tegmentum terminals synapsing on VTA dopaminergic neurons that project to the NAc produces reward-related behavior, whereas activation of the LHb or RMTg terminals within the VTA that modulate dopamine neurons projecting to the mPFC produces aversion (Lammel et al., 2012). This study highlights the topographical input to the VTA and may explain the conflicting reports of whether aversive stimuli excite or inhibit VTA dopaminergic activity.

Other brain structures that either directly or indirectly modulate VTA dopaminergic circuitry are the NAc, amygdala, and the bed nucleus of the stria terminalis (BST). Medium spiny neurons within the NAc are GABAergic neurons that comprise the striatonigral (direct) and striatopallidal (indirect) cortico-basal ganglia pathways. There are two subtypes of medium spiny neurons within the NAc that respond to different patterns of dopaminergic firing patterns (Grace et al., 2007; Schultz, 2007). A burst of phasic firing is responsible for activation of medium spiny neurons containing low-affinity D1 dopamine receptors, substance P, and dynorphin. Activation of these neurons encodes reward-like behavior (Mirenowicz and Schultz, 1994; Grace et al., 2007; Carlezon and Thomas, 2009; Hikida et al., 2010, 2013). They project back to the VTA, synapsing primarily on GABAergic interneurons (Xia et al., 2011) producing a disinhibition that results in excitation of dopaminergic transmission. However, a recent study challenges the exclusive feedback onto only VTA GABAergic interneurons. Using a transgenic mouse that expresses MORs only in D1 medium spiny neurons, Cui et al. (2014) demonstrated that morphine evokes dopamine release in the NAc suggesting that these neurons may also synapse directly on VTA dopaminergic neurons. Medium spiny neurons of the indirect pathway contain D2 dopamine receptors. Slow single spike or tonic firing activates D2 dopamine receptors on medium spiny neurons that co-express enkephalin and produce aversion by modulating VTA circuitry via the ventral pallidum (Mirenowicz and Schultz, 1996; Ungless et al., 2004; Grace et al., 2007; Hikida et al., 2010). As in the VTA, there is some evidence that medium spiny neurons within the NAc may be topographically organized, in that hedonic ‘hot spots’ have been described (Peciña et al., 2006; Richard et al., 2013; reviewed by McCutcheon et al., 2012; Berridge and Kringelbach, 2013). Interestingly, interruption of NAc activity (via lidocaine infusion) reversibly alleviates neuropathic pain (Chang et al., 2014).

The amygdala is involved in a wide array of functions including decision-making, memory, attention and fear. The amygdala is another limbic structure that is thought to attribute affective significance to environmental stimuli by forming a link between brain regions that process sensory information and areas involved in the production of emotional responses. A number of clinical and animal studies have indicated that the amygdala, along with the anterior cingulate cortex, plays a critical role in the processing of affective components of pain (Bie et al., 2011). Hence, excitotoxic lesions of the central amygdaloid nucleus or basolateral amygdaloid nucleus suppress intraplantar formalin-induced aversive responses (Tanimoto et al., 2003; Gao et al., 2004). Glutamatergic transmission within the basolateral amygdala via *N*-methyl-D-aspartate (NMDA) receptors has been shown to play a critical role in these aversive responses. The amygdala sends projections to, among other areas, the hypothalamus, VTA, and the cortex, making it a neuroanatomical structure well positioned to mediate the negative affect (aversiveness) associated with chronic pain (Murray, 2007; Jennings et al., 2013). The extended amygdala includes the BST and the central nucleus of the amygdala. The amygdala modulates the mesolimbic circuitry by sending projections to the NAc and to the BST. There is also evidence for a prominent direct projection from the ventral BST to the VTA (Georges and Aston-Jones, 2001) and local glutamate microinfusion into the ventral BST increased the firing and bursting activity of VTA dopamine neurons (Georges and Aston-Jones, 2002).

## OPIOID RECEPTORS MODULATE PAIN AND REWARD

The opioid system is involved in modulating pain and reward. Opioid receptors are a group of G-protein coupled receptors divided into three families: the MOR, DOR, and KORs. These receptors are activated by three classes of endogenous opioid peptides, beta-endorphin, dynorphin, and enkephalin, that are derived from three precursor peptides (proopiomelanocortin, proenkephalin, and prodynorphin, respectively). The selectivity and distribution of the opioid peptide and receptor systems suggests enkephalin and beta-endorphin act through the MOR and DOR, and dynorphin via the KOR. A fourth opioid receptor family, nociceptin, is distinct from the classical opioid receptor family, in that the endogenous opioid peptides do not bind to it with high affinity (Mollereau et al., 1994). Rather, peptides derived from the pro-orphanin FQ/nociceptin peptide are considered the primary endogenous ligand (Meunier et al., 1995; Reinscheid et al., 1995). Activation of nociceptin receptors opposes the analgesic and rewarding actions of the classical opioid receptors (Mogil et al., 1996; Murphy et al., 1999; Vazquez-DeRose et al., 2013).

The opioid receptors and their peptides are distributed throughout the central and peripheral nervous system in a distinct but overlapping manner (Mansour et al., 1988). The MOR is widely distributed throughout the brainstem, midbrain, and forebrain structures, and mediates most of the analgesia and reinforcing effects of opioid agonists, such as morphine (Kieffer and Gavériaux-Ruff, 2002). DORs, on the other hand, are highly expressed in forebrain regions, including the olfactory bulb, striatum, and cortex (Mansour et al., 1993). Activation of the DOR produces minimal analgesia in acute pain models but develops an analgesic effect in rodent models of chronic pain, where the DOR responses are up-regulated (Cahill et al., 2007; Pradhan et al., 2011). Like the MOR, the DOR positively modulates hedonic state, but to a lesser extent. DOR agonists are anxiolytic (Saitoh and Yamada, 2012), but they are not self-administered and have lower abuse liability than MOR agonists (Negus et al., 1998; Brandt et al., 2001; Stevenson et al., 2005). KOR and MOR expression widely overlaps throughout the brain. However, in contrast to the MOR, activation of the KOR negatively modulates mood and is aversive (Wadenberg, 2003). Systemic KOR agonists also produce robust analgesia (Kolesnikov et al., 1996). KORs are located in the spinal cord and brain stem, and part of their analgesic effect is due to the direct inhibition of pain pathways (Simonin et al., 1995). Recently, we have shown another element of KOR analgesia is a result of their engagement of stress pathways (Taylor et al., 2014). The dynorphin-KOR system plays a central role in the dysphoric elements of stress. Stress induces the release of the opioid peptide dynorphin, an agonist at the KOR, and the aversive effects of stress are mimicked by activation of KORs in various limbic structures in the brain (Knoll and Carlezon, 2010). Dynorphin is released in response to stress via corticotrophin releasing factor (CRF), where it activates KORs in several brain regions involved in affect, including the dorsal raphe nucleus, basal lateral amygdala, hippocampus, and VTA (Nabeshima et al., 1992; Land et al., 2008). Blocking KOR signaling or dynorphin through antibodies or gene disruption blocks stress-induced immobility and produces anti-depressant-like effects (Newton et al., 2002; Mague et al., 2003; Mclaughlin et al., 2003; Shirayama et al., 2004). Further, interfering with KOR signaling blocks the development of avoidance behavior associated with a stressful cue (Land et al., 2008). This suggests the dynorphin/KOR system plays a central role in the aversive stress experience.

While some studies implicate a positive role for the dynorphin/KOR system in anxiety-like behavior (Knoll et al., 2007; Wittmann et al., 2009), other studies have reported that the dynorphin/KOR system decreases anxiety-like behavior (Kudryavtseva et al., 2004; Bilkei-Gorzo et al., 2008). Additionally, transgenic mice with deletion of the KOR show no difference in behavior using a common test of anxiety (elevated plus maze) that is accepted to have predictive validity for pharmacological screening of anxiolytic drugs that reduce anxiety in humans, suggesting a minimal role for KOR in such behaviors (Simonin et al., 1998). Although is not inconceivable that some of these studies are confounded by the side effect profile of KOR agonists, which includes being hallucinogenic (Roth et al., 2002), producing dysphoria (Pfeiffer et al., 1986; Land et al., 2008), and inducing hypo-locomotor activity (Simonin et al., 1998). Nevertheless, salvinorin A, an illicitly used agonist at KORs, is a psychotropic that produces hallucinations, suggesting that activation of KORs may not cause dysphoria in all individuals.

Chronic pain produces anxiety and dysphoria that suggests the engagement of the dynorphin/kappa opioid system (Narita et al., 2006a). In the spinal cord, chronic pain leads to the sustained release of dynorphin, which is hypothesized to be an analgesic response to a sustained pain state (Iadarola et al., 1988; Wagner et al., 1993; Spetea et al., 2002). Inhibiting KOR activation, either through KOR antagonists or in KOR knockout mice, enhanced tactile allodynia after a peripheral nerve lesion (Obara et al., 2003; Xu et al., 2004; Aita et al., 2010). This is in contrast to the results observed in dynorphin knockout mice, in which loss of dynorphin facilitated the return to normal nociceptive baselines after a peripheral nerve lesion (Wang et al., 2001). This is suggestive of a pronociceptive role for dynorphin in chronic pain, and is in contrast to the antinociceptive effects of KOR agonists described above. While the mechanism behind the pronociceptive effects of dynorphin is unknown, intrathecal injection of dynorphin has been reported to have neurotoxic effects and may exacerbate neuronal damage (Walker et al., 1982; Caudle and Isaac, 1988; Long et al., 1988; Sherwood and Askwith, 2009). Xu et al. (2004) hypothesized that sustained release of dynorphin in chronic pain desensitizes KORs. This would reveal the non-opioid mediated pronociceptive effects of dynorphin, and provide a possible explanation for the discrepancy between the results from dynorphin knockout and KOR knockout mice in chronic pain models. Additionally, pain-induced KOR desensitization is supported by the evidence that KOR agonists have a lowered analgesic potency in chronic pain animals (Xu et al., 2004).

In addition to direct effects on neurons, KORs have also been localized to astrocytes, and KOR agonists induce glial activation *in vivo* (Stiene-Martin and Hauser, 1991; Ruzicka et al., 1995; Stiene-Martin et al., 1998; Aita et al., 2010). Chronic pain leads to astrocyte activation in the spinal cord, and glial activation has been identified as a critical mechanism contributing to the sensitization of peripheral afferents leading to chronic pain (Raghavendra et al., 2003). Dynorphin KO animals do not show astrocyte activation after peripheral nerve injury, suggesting the kappa opioid system may act as a critical neuron-glia signal in chronic pain states (Xu et al., 2007). In primary astrocytes, U-69,593, a KOR agonist, produced the same effects as seen in immortalized astrocytes. Another KOR agonist, 2-methoxymethyl-salvinorin B, elicited sustained ERK1/2 activation, which was correlated with increased primary astrocyte proliferation. Proliferative actions of KOR agonists were abolished by either inhibition of ERK1/2, G-protein subunits or β-arrestin 2, suggesting that both G-protein dependent and independent ERK pathways are required for this outcome (McLennan et al., 2008).

While the bulk of studies investigating the contribution of the dynorphin/KOR system in chronic pain have focused on the spinal cord, there is evidence that this system is affected in supraspinal sites as well. Dynorphin is increased in the parietal cortex after spinal cord injury (Abraham et al., 2000). Increased GTPgS binding of KOR-specific ligands in the amygdala of chronic pain animals has also been described (Narita et al., 2006b).

## KOR REGULATION OF MESOLIMBIC CIRCUITRY

The effect of chronic pain on the supraspinal actions of the dynorphin/KOR system, including anxiety and dysphoria, is an area that remains to be studied. Opioid receptors are widely expressed throughout the brain. This expression is highly regulated and varies by cell type, structure, and activity. Each of the three opioid receptor types is differentially expressed uniquely from each other type. As such, the mix of opioid receptor complements of any given structure varies substantially. KORs are widely expressed throughout the brain, spinal cord, and peripheral tissues. KORs are present in many of the major structures involved in pain and addiction processing. High expression levels of KOR have been detected in the VTA, NAc, prefrontal cortex, hippocampus, striatum, amygdala, BST, locus coeruleus, substantia nigra, dorsal raphe nucleus, pedunculopontine nucleus, and hypothalamus of both the rat and human brains (Peckys and Landwehrmeyer, 1999). These brain areas are implicated in the modulation of reward, mood state, and cognitive function. KORs are also expressed at several levels of pain circuitry, including areas such as the dorsal root ganglia, dorsal spinal cord, rostral ventromedial medulla, PAG, sensory thalamus, and the limbic regions. Activation of KORs *in vivo* produces many effects including analgesia, dysphoria, anxiety, depression, water diuresis, corticosteroid elevations, immunomodulation, relapse to cocaine seeking, and decreases in pilocarpine-induced seizure (Bruijnzeel, 2009; Van’t Veer and Carlezon, 2013). KOR agonists have attracted considerable attention for their ability to exert potent analgesic effects without high abuse potential and to antagonize various MOR-mediated actions in the brain, including analgesia, tolerance, reward, and memory processes (Pan, 1998).

Mounting evidence indicates that KORs play a defining role in modulating dopamine transmission. An early PET study identified that glucose metabolism was increased in the NAc and lateral habenular nucleus following peripheral injection of the KOR agonist U-50488 (Ableitner and Herz, 1989). KOR signaling is also able to modulate synaptic transmission of monoamines in a variety of brain structures involved in reward including the VTA and NAc (Margolis et al., 2003, 2005, 2006; Ford et al., 2007). Two microdialysis studies in rats demonstrated that systemic administration of U-50488 and the KOR antagonist nor-BNI decreased and increased dopamine concentrations in the NAc, respectively (Di Chiara and Imperato, 1988; Maisonneuve et al., 1994). Additionally, KOR receptors are present both on dopaminergic neuron cell bodies in the VTA and the presynaptic terminals in the NAc. It has been reported that dopaminergic cell bodies in the VTA expressing KORs selectively project to the prefrontal cortex (Margolis et al., 2006). Here, the authors demonstrated that local injection of a KOR agonist in the VTA of rats selectively inhibited neurons projecting to the prefrontal cortex, and not the NAc. A contradictory study demonstrated, however, that administration of the KOR antagonist U-69539 was able to inhibit NAc projecting neurons from the VTA, whereas met-enkephalin (via MOR or DOR action) inhibited projections to the basolateral amygdala (Ford et al., 2006). It is unclear why there are discrepancies between these two studies, however the topographic organization of VTA neurons involved in reward and aversion may contribute to such differences. Although, biased agonism observed between different KOR agonists may also be an important factor that would explain such discrepancies (Bruchas et al., 2006; Chavkin, 2011; Rives et al., 2012; Negri et al., 2013; Zhou et al., 2013).

Kappa opioid receptors also modulate dopaminergic tone within the NAc. Significant evidence demonstrates that KORs are highly expressed in the both the ventral and dorsal striatum, with the highest concentration in the medial shell of the NAc (Mansour et al., 1996). Further, electron microscopy data has localized the receptors predominantly in synaptic vesicles in axons terminals within the NAc (Meshul and McGinty, 2000). Thus, they are poised to negatively modulate dopamine transmission in this brain region and may serve to affect mood and reward function. Donzanti et al. (1992) demonstrated that application of multiple KOR agonists directly into the NAc inhibited dopamine as measured by microdialysis. In another study, U-50488 was able to inhibit release of dopamine from rat accumbal slices (Heijna et al., 1990).

Both the KOR and its endogenous opioid peptide dynorphin are expressed in the BST and central nucleus of the amygdala (Poulin et al., 2009). The extended amygdala projects to the BST, which plays a critical role in the regulation of anxiety behavior (Walker and Davis, 2008) via release of corticotropin releasing factor (CRF) to enhance glutamate release. GABA is also a transmitter in this projection and it is hypothesized that GABA counteracts the effects of CRF. A recent study demonstrated that the GABAergic transmission is depressed by activation of KORs via a pre-synaptic mechanism within the BST (Li et al., 2012). Thus, CRF and dynorphin release in the extended amygdala act to increase anxiety-like behavior. Indeed, an interaction between CRF and dynorphin is evidenced by the report that anxiogenic effects of stress are encoded by dynorphin in the basolateral amygdala where CRF triggered activation of the dynorphin/KOR system (Bruchas et al., 2009).

Kappa opioid receptors are coupled to heterotrimer Gi/o proteins. Activation of KORs leads to an inhibition of adenylyl cyclase through the Gα subunit and induces increased potassium channel conductance and decreased calcium conductance via the Gβγ subunit. KORs can signal not only through activation of G proteins but also through recruitment of β-arrestins. While β-arrestins are regulatory scaffolding proteins involved in receptor desensitization, they are also signal transducers able to recruit and activate mitogen activated protein kinases (MAPKs). In fact, development of biased agonists for these pharmacological effects has the potential to mitigate some of the side effects associated with KOR activation (Chavkin, 2011). It has been proposed that activation of the MAPK p38 pathway mediates the dysphoric effects produced by selective KOR agonists (Bruchas et al., 2006, 2007). The development of novel KOR agonists that have the potential to be effective analgesics lacking the aversive and dysphoric side effects led to the synthesis of novel small molecule KOR agonists (6′-GNTI, MCKK1-22, triazole and isoquinolinone analogs). These agonists activate the G protein with minimal activity at β-arrestin-MAPK signaling pathway (Rives et al., 2012; Negri et al., 2013; Zhou et al., 2013).

## DO KORs CONTRIBUTE TO PAIN AVERSIVENESS?

What remains unclear, and difficult to ascertain, is whether KORs modulate mesolimbic circuitry and drive the emotional, aversive nature of pain. KOR agonists have dysphoric and psychotomimetic properties in humans and will mediate place aversion in rodents (Shippenberg et al., 1993; Knoll and Carlezon, 2010). These effects can be elicited by direct injection of receptor selective ligands into the VTA (Bals-Kubik et al., 1993). A positive correlation has been demonstrated between dynorphin expression and dysphoria/anhedonia in depressive disorders and withdrawal associated with chronic drug use (Carlezon and Thomas, 2009; Wise and Koob, 2014). Administration of dynorphin and synthetic KOR agonists produces identical anhedonic and dysphoric symptoms characteristic of these disorders (Pfeiffer et al., 1986; Shippenberg and Herz, 1987; Lindholm et al., 2000; Frankel et al., 2008; Isola et al., 2009; Solecki et al., 2009; Knoll and Carlezon, 2010). Non-noxious stressors also activate dynorphin/KORs to produce depressive-like effects that can be blocked by KOR antagonists (Mclaughlin et al., 2003; Chartoff et al., 2009; Bruchas et al., 2010). There is convincing evidence that the aversive properties of KOR agonists are mediated by a negative modulation of the mesolimbic dopamine system (Shippenberg et al., 1993; Chefer et al., 2013), although serotonergic neurons within the dorsal raphe nucleus projecting to the rostral NAc are also proposed to underlie KOR mediated aversion (Land et al., 2009). Further evidence that modulation of serotonergic circuitry contributes to KOR mediated aversion is demonstrated by the observation that serotonin transporter knockout mice do not exhibit KOR-mediated aversion, but restoring this transporter via lentiviral injection in the ventral striatum recovered the pro-depressive effects (Schindler et al., 2012). In contrast, others have reported that KOR agonists continue to produce a place aversion in serotonin transporter knockout mice (Thompson et al., 2013) and that U50,488 produced a hypodopaminergic and hyposerotonergic state in the absence of the serotonin transporter. The observation that selective serotonin re-uptake inhibitor (SSRI) drugs show little efficacy in alleviating chronic pain of various etiologies suggests that serotonin may not be an important monoamine in the aversive component of pain (Moja et al., 2005; Gilron et al., 2006; Sumpton and Moulin, 2014). Nevertheless, activity of medium spiny neurons expressing dopamine receptors within the NAc appears necessary for KOR mediated aversion. Concomitant with altered dopamine transmission, interaction with KORs has been demonstrated to modulate brain reward function, both to natural reward and to drugs of abuse. KOR agonists have been shown to increase food intake in mice and rats, including a direct administration of dynorphin A into the VTA (Hamilton and Bozarth, 1988; Badiani et al., 2001). Though the exact mechanism behind KOR mediated food intake is unclear, it may be a process by which the animal attempts to offset decreased dopamine levels resulting from administration of KOR agonists. Intracranial self-stimulation (ICSS) can be used experimentally to measure alterations in reward thresholds. In one study, the KOR agonist U-69,593 was shown to increase brain reward thresholds for ICSS, indicating a depressive-like state, which was reversed with administration of a KOR antagonist (Todtenkopf et al., 2004). Altered reward states resulting from KOR activation are likely intimately linked with changes in dopamine transmission. For instance, both intra-VTA and intra-NAc administration of U-50488 results in conditioned place aversion in rats (Bals-Kubik et al., 1993). As evidence for a role in altered KOR-driven dopamine transmission in mediating these aversive behaviors, genetic deletion of KORs from dopamine neurons was requisite for systemic KOR agonist place aversion (Chefer et al., 2013). Interestingly, the authors were able to rescue U-69593 mediated place aversion by intra-VTA injection of AAV to re-express KORs on dopamine neurons. Anhedonia and negative affect are also observed in the generation of comorbid mood disorders in neuropathic pain (Yalcin and Barrot, 2014). Thus, KOR modulation of dopamine circuitry and reward may serve as a putative mechanism for mediating the onset of negative emotional states and affect in chronic pain.

Evidence for a role of dynorphin in linking the depression of both behavior and dopaminergic transmission in chronic pain states remains sparse. It is recognized that acute pain (like euphorogenic drugs) activates dopaminergic transmission in brain reward circuitry including the NAc (Boutelle et al., 1990; Scott et al., 2006), whereas chronic or prolonged on-going pain produces the opposite effect (Wood et al., 2007; Geha et al., 2008; Pais-Vieira et al., 2009). Thus, it would be predicted that KOR involvement in modulating pain aversion would occur in chronic pain states where dopamine dysfunction has been described. A recent study demonstrated that CRF is a salient stressor in animal models of chronic pain where either CRF antagonists or CRF-saporin alleviated pain hypersensitivities (Hummel et al., 2010). Stress has been shown to activate the transcription factor CREB (cAMP response element-binding protein) in the NAc, and CREB-mediated increases in dynorphin function in this region contribute to depressive-like behavioral signs including anhedonia in the ICSS test (Pliakas et al., 2001; Chartoff et al., 2009; Muschamp et al., 2011). Additionally, KOR activation in the mPFC causes local reductions in dopamine levels and establishes conditioned place aversions (Tejeda et al., 2013), suggesting that elevated dynorphin function in this region can produce dysphoria. CRF is increased in the limbic system of chronic pain conditions (Rouwette et al., 2012), and injection of CRF into the VTA suppresses dopamine output to the NAc (Wanat et al., 2013). Since KOR antagonists block CRF induced stress responses (Bruchas et al., 2009), it has been hypothesized that KOR may modulate the dysphoric/aversive component of pain via regulation of CRF. However, a recent studies by Leitl et al. (2014a,b) recently reported that KORs are not involved in pain-induced changes in dopamine transmission. Both acute visceral pain (via intraperitoneal injection of lactic acid) and tonic pain (intraplantar injection of formalin) caused reduction in NAc dopamine release and a depression of ICSS, which was not recovered by pretreatment with a KOR antagonist. These studies highlight the influence of pain on dopamine transmission but argue that KOR is not involved in regulation of dopaminergic transmission by an acute or tonic pain stimulus within relatively short time periods. Previous studies demonstrated that KOR activation depressed both ICSS and NAc dopamine release (Todtenkopf et al., 2004; Zhang et al., 2005; Carlezon et al., 2006; Negus et al., 2010). It remains unclear if KORs are not involved in pain modulation of dopaminergic circuitry or if the negative outcome of the Leitl studies (Leitl et al., 2014a,b) was due to study design. The occurrence of anxiety and depressive behaviors that accompany chronic pain states in rodents do not typically begin to manifest until weeks 4 and 6–8 respectively (Yalcin et al., 2011). Thus, the KOR system may only be engaged at later time points following tissue or nerve damage which induces a chronic pain state. Alternatively, KORs may not be critical for the expression of chronic pain but contributes to the modest effects of analgesics in treating some forms of chronic pain including neuropathic pain. Opioid-induced dopamine release in the NAc is attenuated in rodents with neuropathic pain (Ozaki et al., 2002). This result was proposed to explain the lack of opioid addiction in chronic pain. However, an alternative interpretation is that the lack of opioid-induced dopamine release may account for the blunted analgesic properties of opioids in treating this type of pain or in the precipitation of comorbidities such as depression. There is evidence that KORs are responsible for the blunted rewarding effects of opioids induced by a tonic inflammatory pain stimulus. Hence, the effects of morphine induced place preference and morphine induced dopamine release in rats were attenuated by formalin treatment, which was prevented by KOR antagonist pre-treatment (Narita et al., 2005). In line with these results, morphine evoked dopamine release was blunted in the NAc of formalin injected animals, an effect that was reversed with microinjection of an anti-dynorphin antibody in this brain region.

## CONCLUSION

The perception of pain and processes of reward and aversion are complex, multifaceted phenomena manifested through extensive processing in and between multiple brain structures. Of note, these networks exhibit extensive anatomical overlap with several major brain structures are important nodes in pain, pleasure and aversion processing. The mesolimbic system is one point of convergence that lends credence and consilience to the extensive evidence for interactions between pain, reward, and aversion.

The aforementioned studies provide evidence for the role of KOR in modulating dopaminergic neurotransmission in reward circuitry and the influence of dopamine in the transduction and generation of pain processing. Pharmacological manipulation of KOR can be used to modify dopamine transmission and negative affect. An engaging hypothesis holds the upregulation of dynorphin/KOR in chronic pain states to be causal in the generation of concomitant depression and mood disorders. This remains to be fully tested, however supporting evidence includes upregulation of dynorphin following chronic drug use and in post-mortem suicide patients where stress, depression, and anxiety disorders have developed.

There is a clear a role for the dynorphin/KOR system in modulating the interplay of pain and reward processing. Through modulation of limbic neurotransmission, this system produces aversion, stress affect, and depression. The manifestation of these processes as corresponding psychiatric disorders is highly comorbid with chronic pain and suicide is exceedingly prevalent in chronic pain patients. These linked conditions have profound and severely deleterious effects on patients’ quality of life. Despite the implication of the KOR system in this progression, accepted treatments targeting it are lacking, thus manipulation of the KOR system may prove valuable in ameliorating chronic pain-induced negative affect.

## Conflict of Interest Statement

The authors declare that the research was conducted in the absence of any commercial or financial relationships that could be construed as a potential conflict of interest.
